# Unraveling the functional consequences of a novel germline missense mutation (R38C) in the yeast model of succinate dehydrogenase subunit B: insights into neurodegenerative disorders

**DOI:** 10.3389/fnmol.2023.1246842

**Published:** 2023-09-28

**Authors:** Jiatong Zheng, Siru Liu, Dongdong Wang, Linlin Li, Surendra Sarsaiya, Hua Zhou, Heng Cai

**Affiliations:** College of Biotechnology and Pharmaceutical Engineering, Nanjing Tech University, Nanjing, China

**Keywords:** *Saccharomyces cerevisiae*, succinate dehydrogenase, R32C, qRT-PCR, GC-MS, hydrogen peroxide stress

## Abstract

This study explores the implications of a novel germline missense mutation (R38C) in the succinate dehydrogenase (SDH) subunit B, which has been linked to neurodegenerative diseases. The mutation was identified from the SDH mutation database and corresponds to the *SDH2*^R32C^ allele, mirroring the human *SDHB*^R38C^ mutation. By subjecting the mutant yeast model to hydrogen peroxide (H_2_O_2_) stress, simulating oxidative stress, we observed heightened sensitivity to oxidative conditions. Quantitative real-time reverse transcription polymerase chain reaction (qRT-PCR) analysis revealed significant regulation (*p* < 0.05) of genes associated with antioxidant systems and energy metabolism. Through gas chromatography-mass spectrometry (GC-MS) analysis, we examined yeast cell metabolites under oxidative stress, uncovering insights into the potential protective role of o-vanillin. This study elucidates the biological mechanisms underlying cellular oxidative stress responses, offering valuable insights into its repercussions. These findings shed light on innovative avenues for addressing neurodegenerative diseases, potentially revolutionizing therapeutic strategies.

## Introduction

The only enzyme that is shared by both the tricarboxylic acid (TCA) cycle and the electron transport chain is succinate dehydrogenase (SDH), also known as succinate-coenzyme Q reductase (SQR) or Complex II (Yamaguchi et al., 2022). The complex is located within the inner mitochondrial membrane. It has a mitochondrial matrix-oriented and a mitochondrial membrane gap integration domain, with SDHA and SDHB hydrophilic subunits implanted in the inner mitochondrial membrane and SDHC and SDHD hydrophobic subunits positioned on the mitochondrial matrix side (Moosavi et al., 2020).

According to Schweizer et al. (2020), SDHB deficiency, or inadequate succinate dehydrogenase B subunit, is frequently present in paragangliomas (PCPGs). In SDHB mutant PCPGs, the levels of reactive oxygen species (ROS) are elevated, the tricarboxylic cycle is metabolically reprogrammed, and the electron transport chain is altered (Goffrini et al., 2009). Cancer is characterized by oxidative stress, which speeds up the growth of tumors and the spread of the disease (Quinlan et al., 2012; Bezawork-Geleta et al., 2018; Sun et al., 2020). Examples include PCPGs with SDHB mutations and malignancies with IDH1 mutations. Cellular metabolism and redox balance were found to be altered by the functional disruption of mitochondrial complex II brought on by SDHB mutations in cancer cells (Mauer et al., 2020).

Diabetes, cardiovascular disease, and many other illnesses are all linked to oxidative stress (Rhee and Bae, 2015). Numerous studies have shown over time that oxidative stress plays a significant role in the development of illnesses (Zhang et al., 2022). Given that oxidative stress has a role in paragangliomatosis, antioxidant therapy may be employed to treat the disorder (Henderson et al., 2009). However, more fundamental research is required to completely comprehend the mechanisms through which oxidative stress contributes to the development of pheochromocytoma/paraganglioma (PGLs), as these treatments have not yet shown evidence of therapeutic benefit.

Recently, researchers have used yeast models to examine the functional effects of gene mutations on the enzymatic activity and respiratory activity of SDH (Szeto et al., 2007; Beimers et al., 2022). Yeast is a good model for studying gene functions related to human illnesses because it can survive without a working mitochondrial respiratory chain (Baruffini and Lodi, 2010; Invernizzi et al., 2013; Abugable et al., 2017; Prevost et al., 2018; Basu et al., 2020; Gilea et al., 2021). The ability of yeast cells to quickly delete, modify, and reinsert *S. cerevisiae* genes, in contrast to human cells, provides a wealth of important information for understanding the molecular causes of disease (Kumar and Reichert, 2021). This study uses the *SDH2* gene, which is the yeast equivalent of the mammalian SDHB gene, as a model for a typical family of PGL mutations.

The TCA Circulating Gene Mutation Database^1^ provides an overview of complex II mutations (Hoekstra and Bayley, 2013). Around 15% of paragangliomas are thought to be caused by all Complex II mutations, which are thought to have tumor-suppressive roles (Pęczkowska et al., 2013; Richter et al., 2019; Wurth et al., 2021). The reported number of mutations for each component gene varies significantly for unknown reasons. Five categories of gene mutations exist: harmful, potentially pathogenic, unknown clinical significance (VUS), potentially benign, and benign. Some diseases are brought on by a gene mutation that can be seen in the clinical manifestation. The newly identified mutation, on the other hand, is typically not supported by any conclusive literature and is indicated in the disease mutation database as having questionable clinical relevance. Since the mutation with unknown clinical implications may be pathogenic, numerous functional investigations can be done to demonstrate whether the mutation is deleterious at this time (Makhnoon et al., 2019).

In the study presented here, the R38C missense mutation in the B subunit of a new kind of succinate dehydrogenase (SDH) linked to neurodegenerative illnesses is examined for its functional effects. To our knowledge, this particular mutation has not been previously described, and it is yet unclear how it affects cellular function and disease pathogenesis. Our research intends to close this information gap and illuminate the molecular significance of this mutation in relation to neurodegenerative disorders.

**Figure 1:**
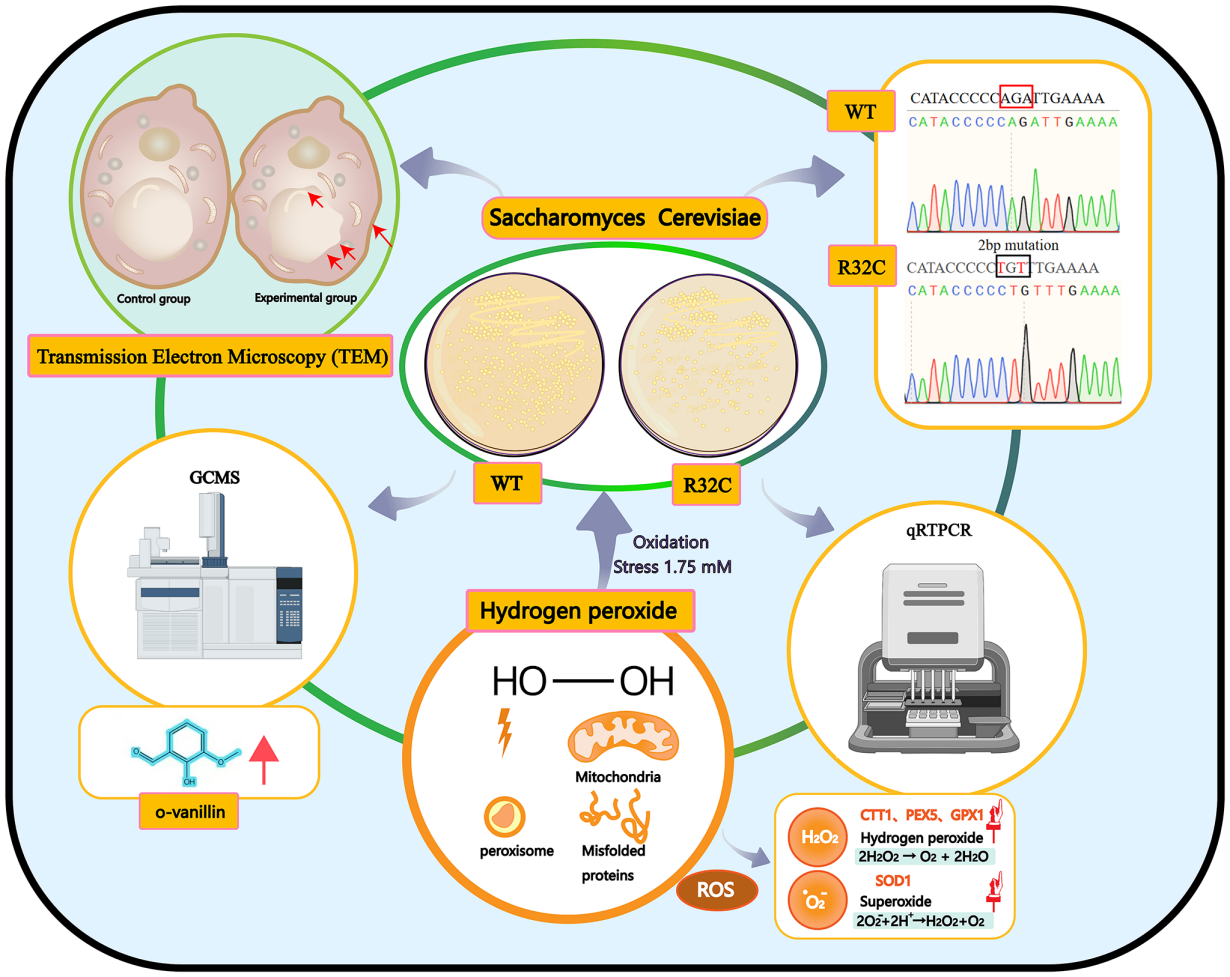
Graphical summary of the methods and results used in this study. Schematic representation of the interactions of the effects of hydrogen peroxide stress on *Saccharomyces cerevisiae*. Production of peroxisomes and misfolded proteins under oxidative stress.

In this work, phenotypic changes of yeast cell development were evaluated first. The internal ultrastructure of yeast cells was then described at the organelle level using scanning TEM. The molecular expression of genes related to energy metabolism and antioxidant enzyme systems was examined using quantitative real-time reverse transcription polymerase chain reaction (qRT-PCR). The metabolites of yeast cells under oxidative stress were then analyzed using GC-MS. This study seeks to provide a novel perspective on the molecular mechanisms affecting metabolites and yeast cell resistance to oxidative stress caused by SDH2 mutation (Figure 1).

## Materials and methods

### Strains and growth conditions

The strain used in this study was *Saccharomyces cerevisiae* wild type BY4741 (MATa *his3Δ1 leu2Δ0 met15Δ0 ura3Δ0*). The YPD medium (1% yeast extract, 2% peptone, and 2% glucose) was used to culture the cells. To check for *SDH2* transformants, cells were cultured in a YPD medium with 200 μg/mL genomycin as an addition. To test the sensitivity under oxidative conditions, different concentrations of hydrogen peroxide were applied to YPD medium. Cells were incubated in a liquid YPD medium while being agitated at 220 rpm and 29°C for other tests.

### Knockout of plasmids and target genes

As previously stated (Lopez-Berges et al., 2010), our work used the plasmid pUG6 and the primers specified in Table 1 to replace a particular gene by PCR-mediated homologous integration. Geneticin resistance (KanMX) was used as a screenable marker. Using knockout primers, a 1.8 kb DNA fragment from the pUG6 genome was amplified by PCR to produce a knockout cassette for SDH2. A genomic analysis of the transformed strain was conducted using PCR to verify the insertion of outside genes into the structure.

**Table 1:** Primers used in this study.

Primer	Sequence (5′→3′)
QC-F	TGTTAGGCTAAAGGAAGGGATTGAAAGGAATATAGTTGAGCTATACAGCTGAAGCTTCGTACGC
QC-R	TCATAGAGAGTAAACATAAACGCCTAACGTATAGGAATGTGTGAGGCATAGGCCACTAGTGGATCTG
Yz-a	CTTCATTCCCATCAGCG
B	CAGCCAGTTTAGTCTGACCATCT
C	CCTCGACATCATCTGCCCAG
Yz-d	CTATGCCACTCCTGGTAAG

### One-step cloning method to construct mutant strains

Table 2 contains a list of the investigation’s primers, and PYX212 was the plasmid used in this study. Using the BY4741 genome as a template, SDH2-F and SDH2-R were used as primers for PCR amplification of the SDH2 fragment with the enzymatic cut site (Tao et al., 2020; Liang et al., 2021).

**Table 2:** Primers used in this study.

Primer	Sequence (5′→3′)
SDH2-F	CCCAAGCTTATGTTGAACGTGCTATTGAGA
SDH2-R	CGAGCTCCTAGGCAAATGCCAAAGATTT
R32C-F	TTTACGGTTGGAATCCAGACGAG
R32C-R	GGATTCCAACCGTAAACTTTAAAAGT
R32D-F	ACCCCCGATTTGAAAACTTTTAAAGTT
R32D-R	TTCAAATCGGGGGTATGCGTAGC
R32S-F	ACCCCCTCTTTGAAAACTTTTAAAGTT
R32S-R	TTCAAAGAGGGGGTATGCGTAGC
R-1	TGAACGTGCTATTGAGAAGGA
R-2	TGTGCTGCAAGGCGATTA

### Detection of growth phenotypes and growth curves of *Saccharomyces cerevisiae* under different concentrations of hydrogen peroxide stress

To determine *S. cerevisiae* cells’ receptivity to oxidative stress, they were grown for a whole day at 29°C in a YPD liquid medium. The cells were suspended in fresh YPD liquid the following day with an OD_600_ value set to 1. Before being spotted in 3 μL onto the control and 1, 1.5, and 1.75 mM H_2_O_2_ YPD solid medium and incubated upside down at 30°C for 2–3 d, the samples were diluted to 10^−1^, 10^−2^, 10^−3^, 10^−4^, and 10^−5^. The growth was observed, noted, and visually documented. To investigate the growth inhibition of BY4741 and mutant strains under oxidative stress, cells were grown in a liquid medium containing YPD, and 1, 1.5, and 1.75 mM H_2_O_2_ and OD_600_ values were checked every 3 h. Data were acquired in order to plot growth curves (Edwards et al., 2020).

### MTT assay

In order to perform the MTT experiment, yeast cells were grown to the logarithmic growth stage, treated with hydrogen peroxide at doses of 0 and 1.75 mM, respectively. Each well received a total of 20 μL of a 5 mg/mL MTT solution (Biyun Tian, China). The supernatant was removed by centrifugation after 4 h of incubation, and 200 μL of dimethyl sulfoxide was then added. The culture plate was placed on an enzyme marker (Thermo, United States) and the optical density (OD) value was determined at 490 nm after shaking for 10 min.

### Cellular ROS assays

2′,7′-Dichlorofluorescein diacetate (DCFH-DA, Molecular Probes, United States) was used to quantify ROS levels (Serpa et al., 2021). Overnight, strains were grown in YPD medium. After being collected, the cells were diluted in fresh YPD to an OD_600_ of 0.5, and then incubated for 6 h at 30°C. One hundred and eighty revolutions per minute, then 1.75 mM of hydrogen peroxide was added. The cells were then exposed to hydrogen peroxide for one additional hour. Control and treated cells were stained with 20 mg/mL DCFH-DA and then incubated at 30°C for 30 min after being washed with phosphate free saline (PBS). Following a second PBS buffer wash, cells were examined with a fluorescent enzyme marker (Abdullah et al., 2022).

### Determination of enzyme activity of antioxidant system, SDH enzyme activity and degree of lipid peroxidation

The level of lipid peroxidation and cellular activity in the cells were determined using the malondialdehyde (MDA) assay kit, superoxide dismutase (SOD), glutathione GPx, and succinate dehydrogenase (SDH) assay kits (Nanjing Jiancheng Bioengineering Institute), respectively (V et al., 2022).

### Construction of dual expression vectors for yeast protein models

Using pESC-URA plasmid, SDH2-R32C and *SNCA* genes (synthesized by Kingsley Biotechnology Co., Ltd., China) were exogenously inserted and introduced into yeast cells for expression (Khulape et al., 2015).

### RNA extraction and quantitative real-time reverse transcription polymerase chain reaction

Regarding the aforementioned technique, cells were given a hydrogen peroxide treatment. Cells under oxidative stress and under control were collected, split using liquid nitrogen, and extracted with RNAiso Plus (TaKaRa) for RNA extraction. With the help of the Taq Pro Universal SYBR qPCR Master Mix from Vazyme Biotechnology in China, real-time fluorescence quantitative PCR was carried out (Qian et al., 2021; Wang et al., 2023). Table 3 provides a list of the primers employed in qRT-PCR tests.

**Table 3:** Primers used in this study.

Primer	Sequence (5′→3′)
ACT1-F	ACTTTCAACGTTCCAGCCTTC
ACT1-R	CGTAAATTGGAACGACGACGTGAGTA
SOD1-F	TGTCAAGTTCGAACAGGCTTCCGA
SOD1-R	AAGGATTGAAGTGAGGACCAGCAG
CTT1-F	ATAACGTTGTTTGCCACGCTTGTA
CTT1-R	TTCAAGGTCAACAGGTTCCCAAGG
PEX5-F	TACAGACTTCAGCAAACCCAACCC
PEX5-R	AAACCGGCTTGATGCGGTTGTTGA
GPX1-F	CGGGCAAAAGCAAGATCCCGTCTAC
GPX1-R	ACCACCTTCCCATTTCGGTCTACC
Pos5p-F	TCCCTAAAGTTACAGAGCGGCTCG
Pos5p-R	GGAATTTGGTGGGGACACGAAATC
SDH1-F	AACAGCAGCCATTTTTGCTGGTGT
SDH1-R	TAATGCCTCACCATTCCACTTCGT
COX4-F	GGTCCTGGTGCTAAAGAGGGTACC
COX4-R	TTCATGGTACCCTTCCTGGACGAA
IDP2-F	AAGGCGAGTTGAGGCTTGTT
IDP2-R	CAAAGGAGGCCTTCGCAAAC
ACO1-F	TGGTGTTGACACCTTCTCCG
ACO1-R	TACCACGACCAGTTGCTTCC

### Transmission electron microscopy

After being treated with hydrogen peroxide, yeast was examined using transmission electron microscopy, as previously mentioned (Bayley et al., 2005). In a nutshell, cells were isolated by centrifugation, twice-washed with PBS, fixed with 2.5% glutaraldehyde at 4°C overnight, followed by 1% osmium acid solution for 1–2 h. Using an ethanol gradient, cells were dehydrated, and TEM analysis was performed (Ubhe, 2023).

### Metabolite analysis

The metabolites in *S. cerevisiae* were identified via gas chromatography–mass spectrometry (GC-MS). For metabolomics analysis, samples were quenched, wall-breaking was eliminated, and they were derivatized. The sample was separated at a controlled flow rate of 1 mL/min using helium with a purity of 99.999% as the carrier gas and a temperature program of 60°C for 1 min, followed by a continuous temperature increase of 12°C/min to 240°C and a target temperature increase of 40°C/min to 320°C for 5 min. The sample was injected at a 4:1 ratio of 1 L with a solvent delay time of 5 min at an input temperature of 250°C and an electron impact (EI) off-source temperature of 230°C. 70 V was utilized as the EI voltage. In full scan mode, data were collected for mass-to-charge ratios (m/z) between 50 and 600 (Liu et al., 2020; Roberts et al., 2023; Tufariello et al., 2023).

### Treatment of cells with o-vanillin

Survival data under oxidative stress conditions were determined using the MTT assay in the presence and absence of treatment with 50 μM o-vanillin, respectively.

### Statistical analysis

The experiments were conducted in triplicate, and the results were analyzed using one-way and two-way ANOVA (Analysis of Variance) to determine their significance. The data is presented in bar graphs, showing the mean and standard deviation of the results, which demonstrate that the experiments were statistically significant.

## Results

### Construction and validation of *Saccharomyces cerevisiae* R32C mutant strain

**Figure 2:**
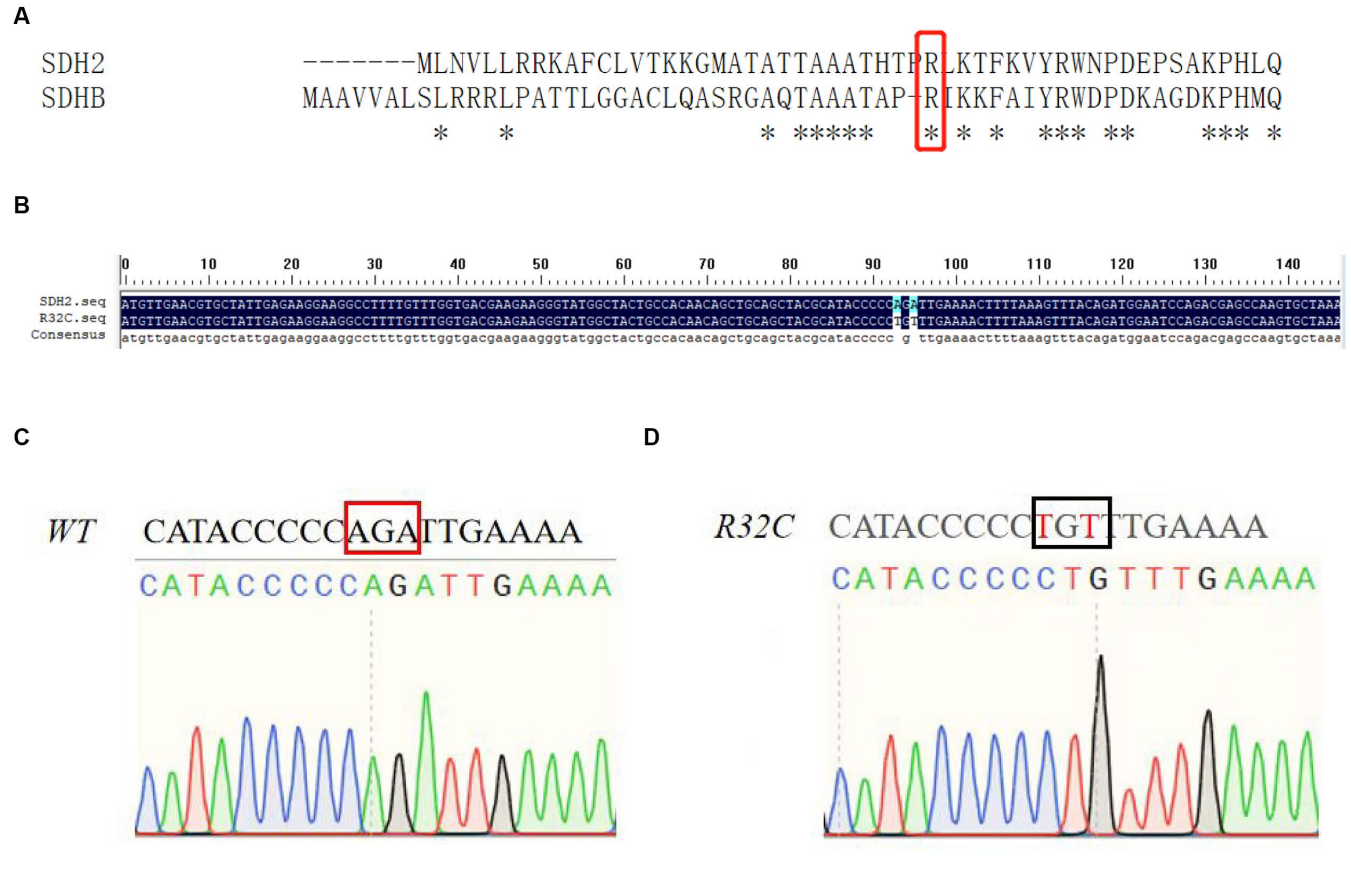
Mutation analysis. **(A)** Sequence alignment of yeast *SDH2* gene with human *SDHB* gene; **(B)** Base sequence alignment of yeast *SDH2* gene with R32C mutant bacteria. This indicates that the residue is conserved; **(C, D)** Sequencing map of yeast *SDH2* wild type gene with R32C mutant bacteria.

The missense mutation c.112C>T, in which the base C at position 112 is transformed to base T, correlating to the amino acid alteration of arginine to cysteine at position 38, was found to be a novel mutation in the *SDHB* gene. This mutation was categorized as having a clinically unknown clinical significance (VUS) mutation. According to the results of the sequence comparison and the preferred codons, the yeast *SDH2* gene differs from the human *SDHB* gene in that it contains base mutations at positions 32 and A, as shown in Figure 2A. Figure 2B illustrates that this residue is conserved. Figures 2C,D show the sequencing profile before and after the base mutation.

### Cellular characterization of the yeast SDH2 mutation (R32C)

**Figure 3:**
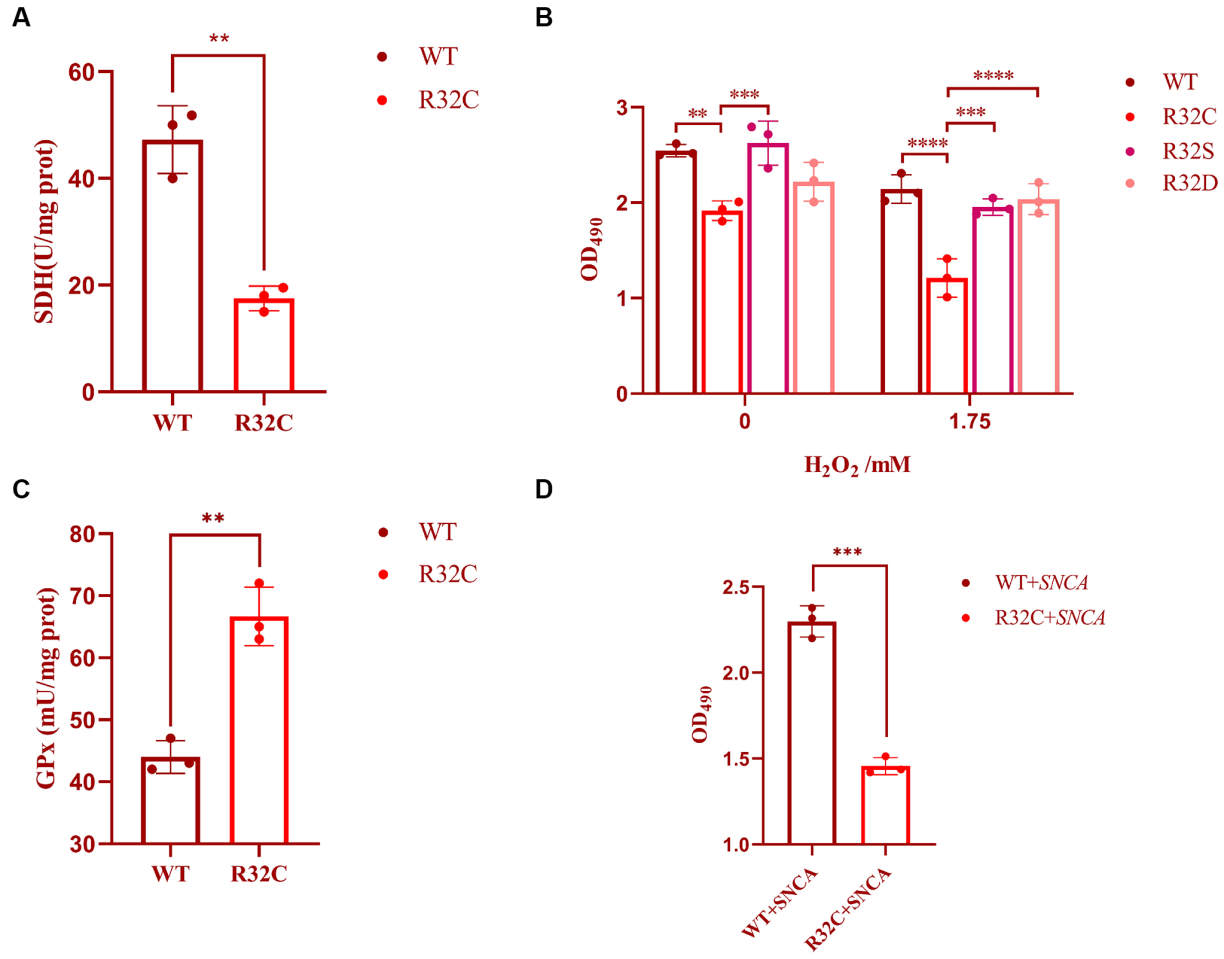
Cellular characterization of SDH2 and R32C. **(A)** Determination of succinate dehydrogenase (SDH) activity by *Saccharomyces cerevisiae* WT and R32C. **(B)** Determination of survival data of WT, R32C, R32S, and R32D by treatment with different concentrations of hydrogen peroxide. **(C)** Determination of WT and R32C glutathione peroxidase (GPx). **(D)** Determination of survival of WT and R32C after introduction of *SNCA*, respectively (mean ± sd). 0.01 < *p* < 0.05 (**); *p* < 0.01 (***); *p* < 0.0001 (****).

The R32C mutation results in changes in the structure or function of the active site, which in turn affects SDH2 activity. The effect of the R32C mutant on the active site was assessed using a succinate dehydrogenase activity assay. The results show that the R32C SDH enzyme activity is lower than that of WT (Figure 3A).

**Figure 4:**
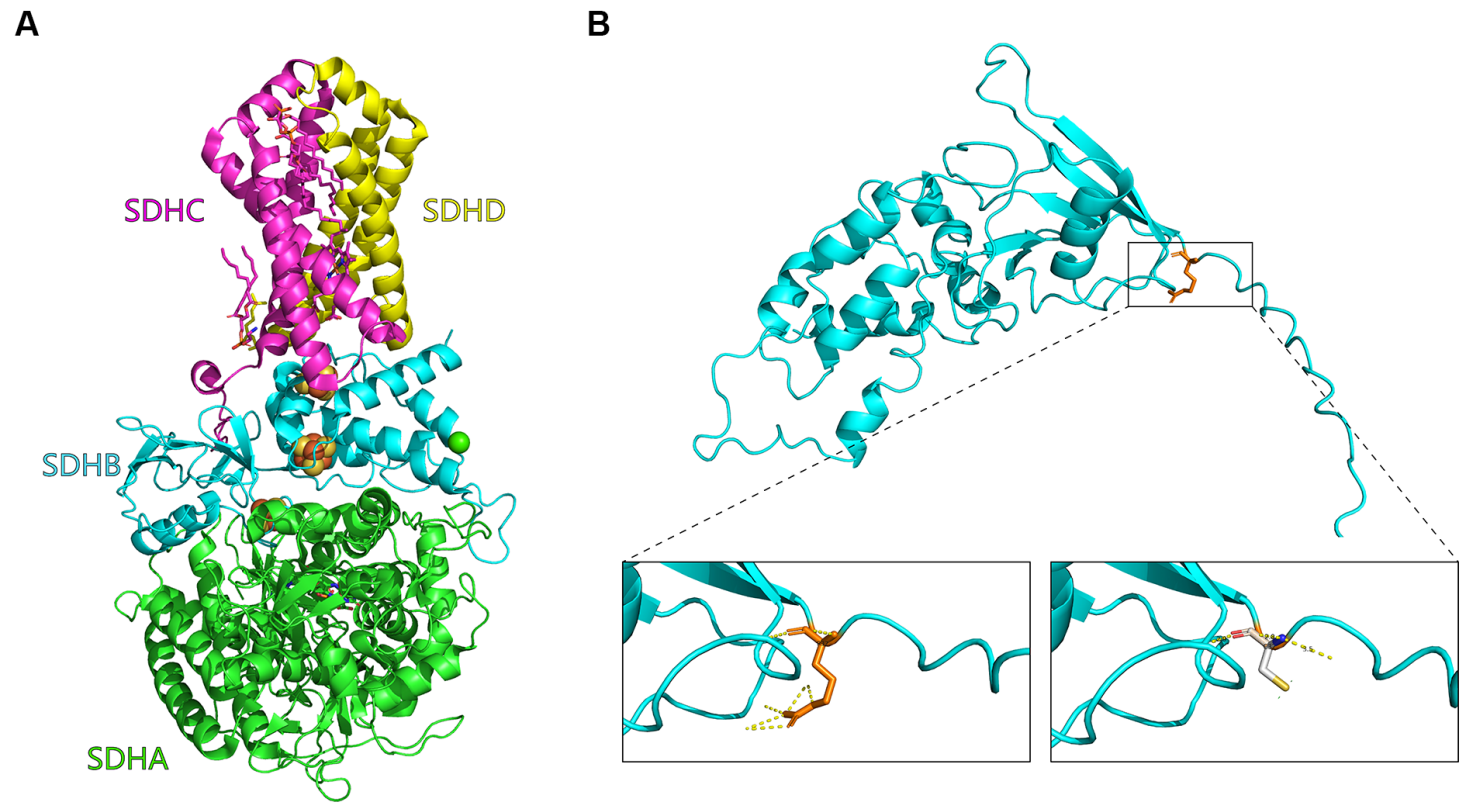
Mutant SDH2 subunit model. **(A)** Three-dimensional structural diagram of succinate dehydrogenase (1NEK.pdb) constructed with PyMOL. the SDHA, SDHB, SDHC, and SDHD subunits are indicated in green, cyan, purple, and yellow, respectively. **(B)** Enlarged view of the *Saccharomyces cerevisiae* SDH2 gene before and after the R32C mutation. On the left is the arginine (R32, orange) of the yeast SDH2 subunit before mutation, and interactions with other amino acids are indicated by yellow dashed lines. On the right is modeling of the mutation of arginine to cysteine on the yeast SDH2 subunit (C32, white).

The R32C mutation changes an amino acid residue from arginine (R) to cysteine (C). Construction of a three-dimensional structural model of the mutant SDH2 subunit protein suggests that this amino acid sequence change alters the normal interactions between SDH2 and its complex partners, resulting in altered function or impaired protein–protein interactions (Figure 4).

### Specificity of the R32C mutation

The R32C mutation leads to an additional cysteine residue in the SDH2 protein. Cysteine residues can be oxidized and are known as redox switches. Other SDHB mutations recapitulated in yeast were not all reported to be sensitive to oxidative stress (Panizza et al., 2013), so this is something more specific to the mutation.

In this study, the effect of substitution on oxidative stress sensitivity was assessed by replacing cysteine with serine (S) and aspartic (D) acids, and cell survival was measured using MTT. The experimental results showed that the survival rate of R32C was significantly lower than that of WT and R32S and R32D under both 0 and 1.75 mM hydrogen peroxide stress (*p* < 0.05), while the cell survival of R32S and R32D was not significantly different from that of WT (*p* > 0.05, Figure 3B).

### Scavenging capacity of WT and R32C for H_2_O_2_

The effect of R32C on intracellular oxidative stress is interesting, so we investigated the intrinsic effects of R32C without added hydrogen peroxide. The effect of H_2_O_2_ scavenging can be assessed indirectly by measuring glutathione peroxidase (GPx). As shown in Figure 3C, the enzyme activity of GPx was significantly higher in R32C compared to WT. Therefore, R32C has an enhanced ability to scavenge H_2_O_2_ and maintained cellular homeostasis by increasing GPx enzyme activity.

### Yeast protein models of neurodegenerative diseases

To study the link between neurodegeneration and R32C mutants, alpha-synuclein (encoded by the *SNCA* gene) was used in yeast to screen R32C mutants for increased sensitivity to the protein. The results of the experiments are shown in Figure 3D. After introducing the *SNCA* gene in WT and R32C, respectively, the MTT method was used to compare the growth survival rates of the two strains, and it was found that the R32C mutant showed higher sensitivity compared to the WT strain, which may indicate a potential link between the mutant and neurodegeneration.

### Hydrogen peroxide disrupts the growth and internal ultrastructure of the R32C mutant strain

**Figure 5:**
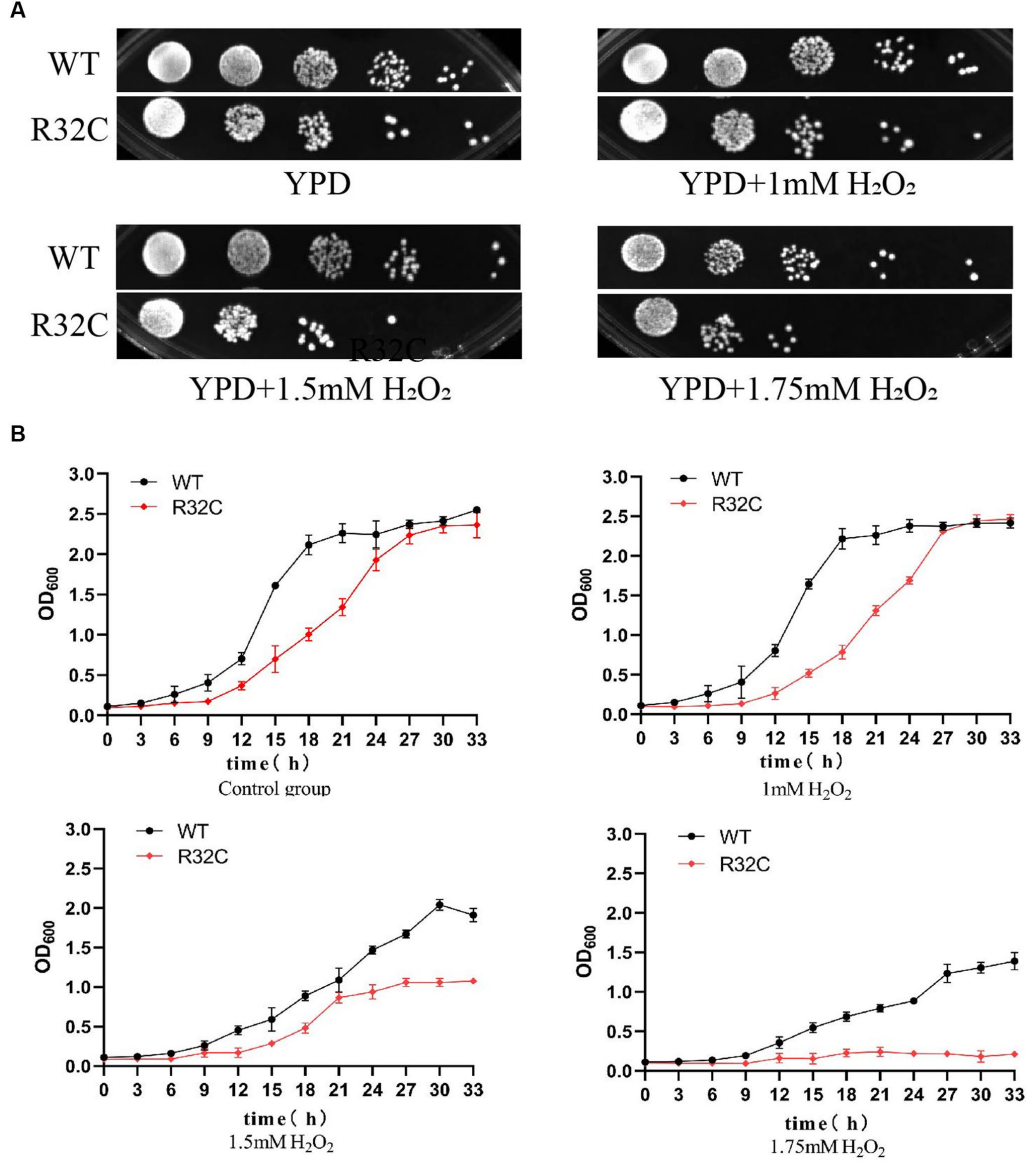
The R32C mutant strain resulted in increased susceptibility to oxidative stress. **(A)** Wild-type strain BY4741 and mutant strain R32C were cultured on solid YPD medium containing 0 mM, 1 mM, 1.5 mM, and 1.75 mM H_2_O_2_. **(B)** OD_600_ was measured at different H_2_O_2_ concentrations to compare the growth inhibition of BY4741 and R32C.

**Figure 6:**
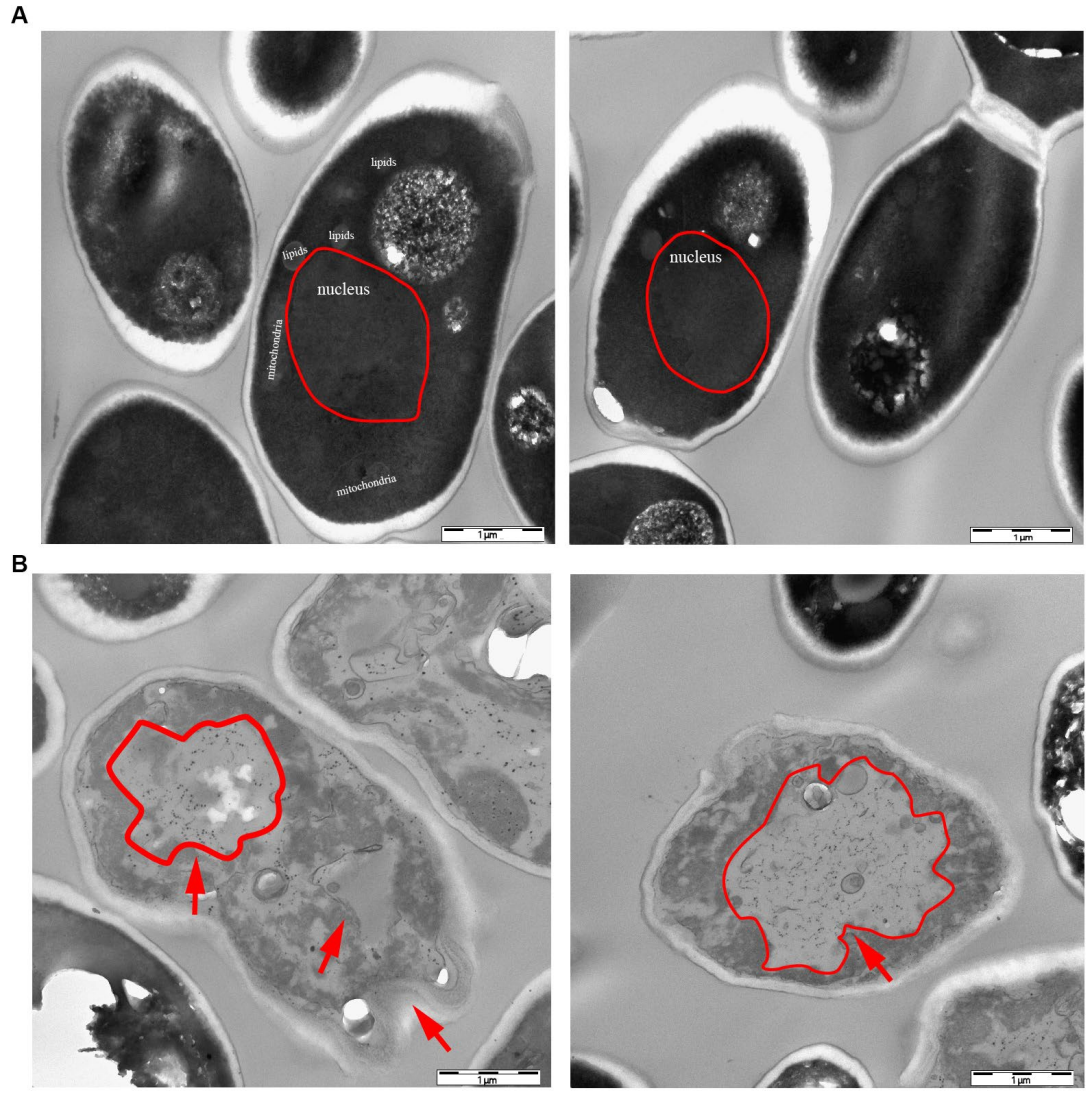
Transmission electron microscopy (TEM) characterization of yeast morphology. **(A)** WT control group without hydrogen peroxide stress. **(B)** Phenotypic characteristics of strain R32C under 1.75 mM hydrogen peroxide stress. As shown by the red arrows, in the hydrogen peroxide-treated cells, the cell morphology appears to be concave and atrophied.

According to the dot plate experiment results, BY4741 was less impacted. At the same time, R32C growth was considerably hindered at 1.75 mM H_2_O_2_ stress (Figure 5A). Based on the results of growth curve experiments, R32C’s growth was found to be inhibited at 1.5 mM H_2_O_2_ stress, although BY4741’s growth was less impacted. Both strains’ growth was inhibited at 1.75 mM H_2_O_2_ stress (Figure 5B). The normal cells exhibited an intact cell wall structure, a clear cell membrane, an evident nucleus and vacuole, and mitochondria were visible in the cell pulp in the absence of further hydrogen peroxide stress (Figure 6A). However, in the hydrogen peroxide-treated cells, the cell morphology appeared depressed and atrophied, the typical cell wall and cell membrane structure vanished, the gap between the cell wall and cytoplasm expanded, the cytoplasm atrophied, electron-dense particles appeared in the middle electron-sparing layer, the contents of the cytoplasm were disorganized, and some of the contents were seen to leak out (Figure 6B). It is shown that oxidative stress causes cellular damage in yeast and its mutant strains, which may be related to the development of neurodegenerative diseases.

### Oxidative damage of R32C due to hydrogen peroxide stress

**Figure 7:**
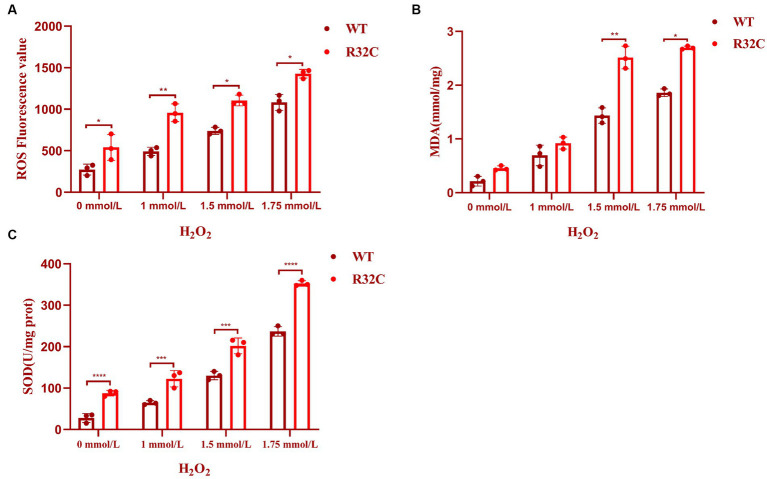
R32C mutation causes oxidative damage to the oxidative system. **(A)** Measurement of intracellular ROS content in *Saccharomyces cerevisiae* under different concentrations of H_2_O_2_ treatment; **(B)** Measurement of intracellular MDA content in *Saccharomyces cerevisiae* under different concentrations of H2O2 treatment. **(C)** Determination of intracellular SOD content of *Saccharomyces cerevisiae* under different concentrations of H_2_O_2_ treatment (mean ± sd). *p* < 0.05(*); 0.01 < *p* < 0.05(**); *p* < 0.01(***); *p* < 0.0001 (****).

An essential indicator of oxidative stress in living things is ROS. MDA is a significant marker in the lipid peroxidation response, which can indirectly represent the degree of free radical damage that cells have been exposed to Wu et al. (2023) and impact the structure of cell membranes (Tsikas, 2017). Hydrogen peroxide stress up-regulated the intracellular ROS content of *S. cerevisiae* and its mutant strains (*p* < 0.05) (Figure 7A) and increased the MDA content at a later stage (*p* < 0.05) (Figure 7B). These preliminary results indicated that lipid peroxidation of yeast cell membranes was exacerbated under oxidative stress.

### R32C antioxidant system damage due to different concentrations of H_2_O_2_ stress

A typical family of biological enzymes called superoxide dismutase plays a critical function in the oxidative and antioxidant balance of the body and can protect cells by scavenging superoxide anion free radicals. As seen in Figure 7C, the SOD enzyme activity increased in wild-type BY4741 and R32C as the concentration of hydrogen peroxide stress rose, demonstrating that hydrogen peroxide stress would enhance the intracellular oxidative stress response and harm the antioxidant system. Additionally, at 1.75 mM H_2_O_2_, the R32C mutant strain exhibited the maximum enzyme activity.

### Different concentrations of H_2_O_2_ stress lead to increased transcript levels of genes related to oxidative stress and antioxidants in *Saccharomyces cerevisiae*

**Figure 8:**
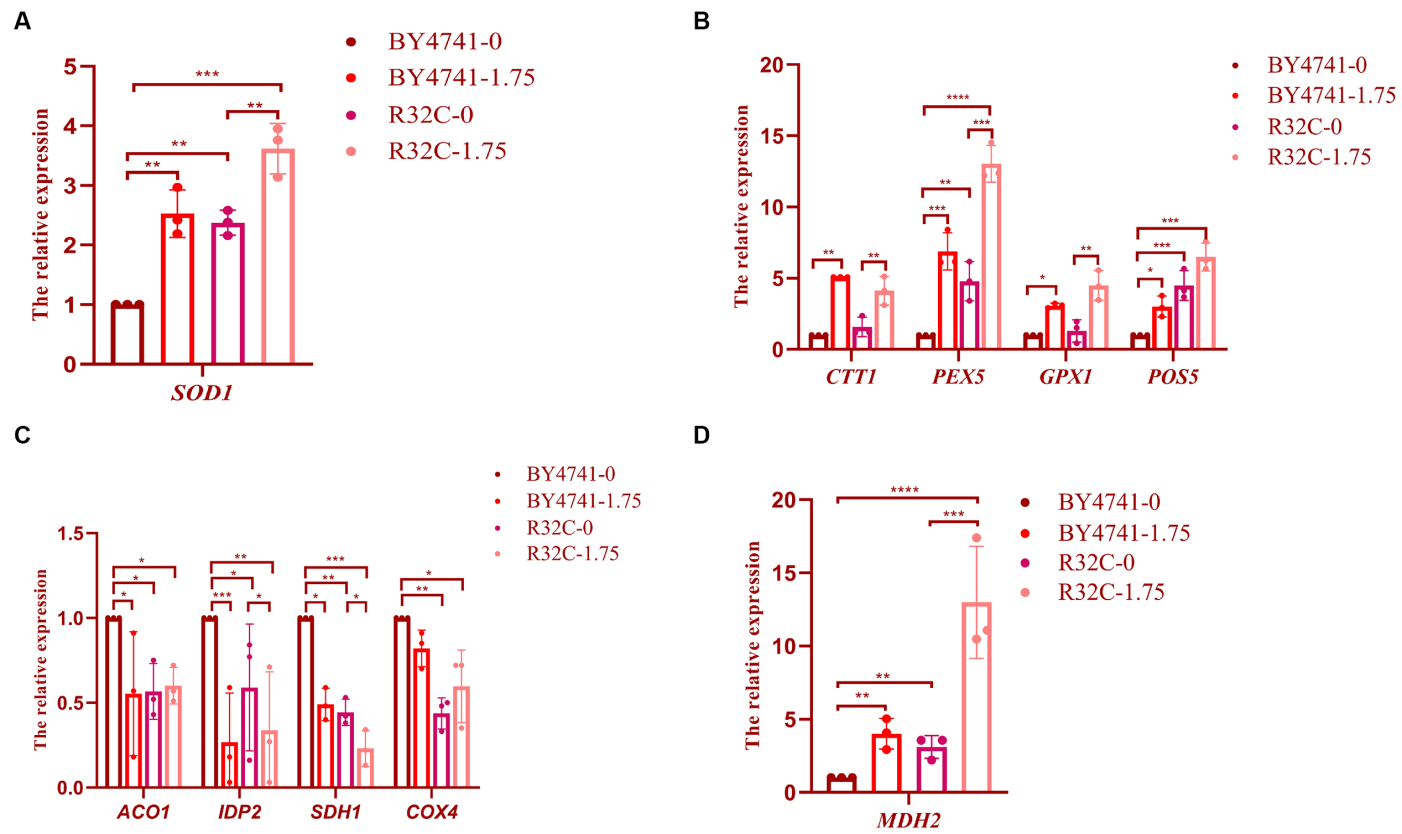
Susceptibility of R32C mutant strain to oxidative stress associated with antioxidant system and energy metabolism. Real-time fluorescence quantitative PCR assay **(A)**: *SOD1* gene transcript levels (Note: BY4741-0, i.e., BY4741 normal culture; BY4741-1.75, i.e., BY4741 cultured at 1.75 mM H_2_O_2_, as described below), **(B)** *CTT1*, *PEX5*, *GPX1*, *POS5* gene transcript levels. **(C)** *ACO1*, *IDP2*, *SDH1*, *COX*4 gene transcription levels. **(D)** *MDH2* gene transcription levels. Data presented is the results of three independent experiments (mean ± sd). *p* < 0.05(*); 0.01 < *p* < 0.05(**); *p* < 0.01(***); *p* < 0.0001(****).

A few genes with antioxidant activity must be expressed in the organism to prevent oxidative damage because cells exposed to high amounts of hydrogen peroxide produce more ROS. To ascertain the relative expression levels of genes linked to oxidative stress and antioxidant, we performed culture in wild-type BY4741 and R32C mutant strains with or without the addition of hydrogen peroxide. The findings demonstrated that the hydrogen peroxide-treated strains had greater levels of *SOD1* gene expression than the untreated strains (Figure 8A). Since hydrogen peroxide stress increases intracellular ROS, it is known that the transcript levels of *SOD1* were elevated in the hydrogen peroxide-treated medium, indicating that intracellular oxidative stress is more significant and that more SOD is needed in the organism to remove oxidative stress.

When the expression levels of the *CTT1, PEX5, GPX1*, and *POS5* genes were compared to those without H_2_O_2_ treatment, the transcript levels of *CTT1* were considerably higher, as shown in Figure 8B. The *CTT1* gene may be able to withstand oxidative damage due to its high expression. While the expression level of the *GPX1* gene did not change significantly, the expression levels of the *PEX5* and *POS5* genes were significantly higher in the R32C mutant compared to BY4741 under normal circumstances, indicating that the level of oxidative stress was significantly increased in the mutant cells. After exposure to H_2_O_2_, the transcript levels of the *PEX5*, *GPX1*, and *POS5* genes rose. The transcript level of the *PEX5* gene increased 14.5-fold when compared to WT, demonstrating that *PEX5* is a crucial gene in the antioxidant pathway.

During oxidative stress, cells undergo various changes to mitigate the damaging effects. For instance, previous studies have reported that the peroxisomal protein *PEX5* plays a crucial role in maintaining peroxisome functionality and protecting against oxidative stress (Wang and Subramani, 2017). *PEX5* is involved in the import of matrix proteins into peroxisomes and has been shown to be upregulated in response to oxidative stress conditions (Walton et al., 2017).

### Different concentrations of H_2_O_2_ stress resulted in the down-regulation of transcript levels of genes related to energy metabolism and the up-regulation of *MDH2* genes in *Saccharomyces cerevisiae*

We used real-time fluorescence quantitative PCR analysis on the expression of important essential genes to clarify the resistance mechanism of hydrogen peroxide stress on the respiratory energy metabolism of yeast. After H_2_O_2_ therapy, the relative expression of the genes *ACO1*, *IDP2*, *SDH*1, and *COX4* was much lower than it was in the group that received no treatment (Figure 8C). Malate dehydrogenase, which is encoded by the *MDH2* gene, showed the most substantial up-regulation of expression (Figure 8D), demonstrating that H_2_O_2_ may efficiently control energy metabolism in *S. cerevisiae* (Supplementary Figure S1).

### Potential protective effect of o-vanillin on yeast under oxidative stress conditions

**Figure 9:**
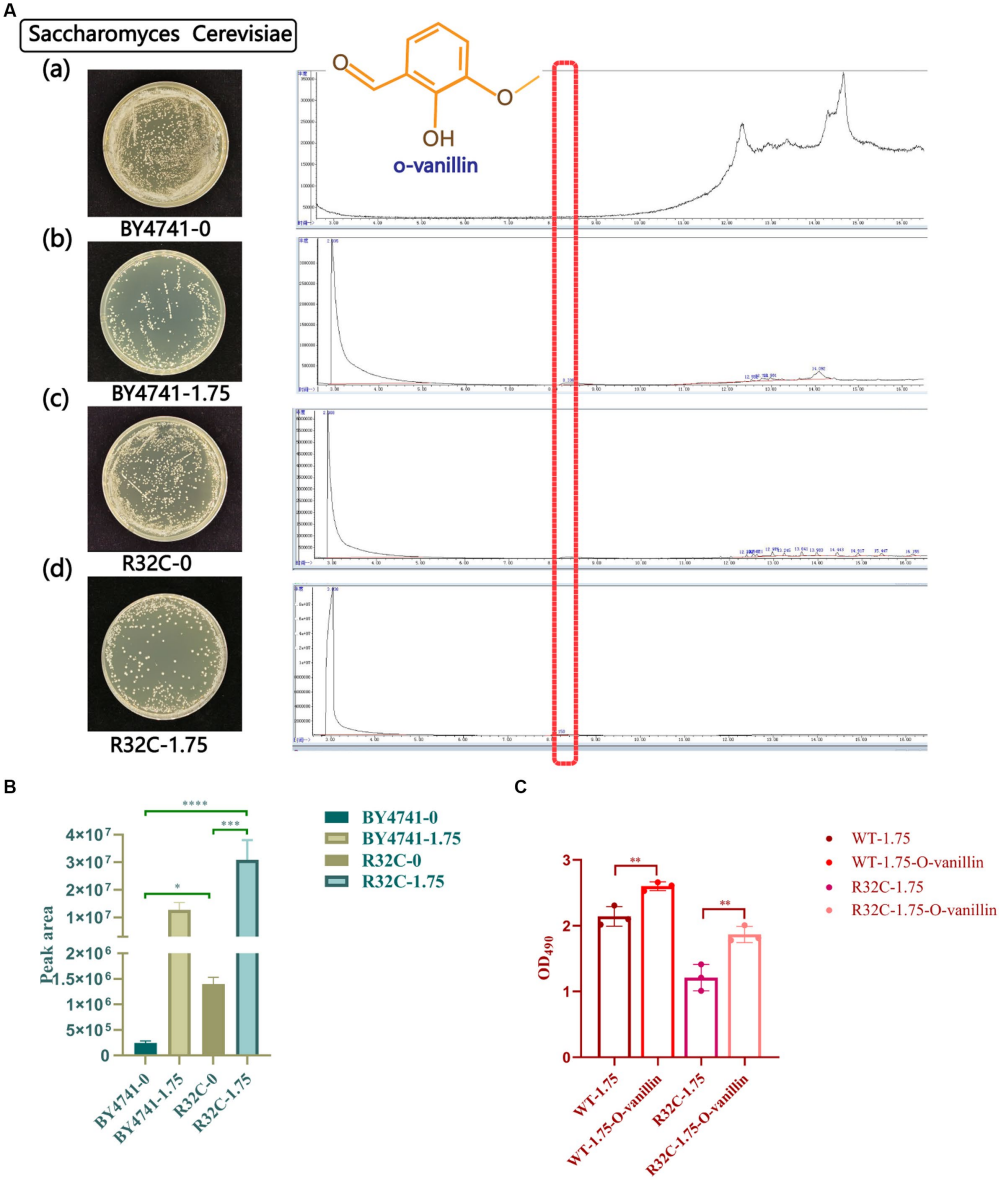
Gas chromatography–mass spectrometry (GC-MS) scans of metabolite profiles of wild-type and mutant yeast strains under hydrogen peroxide stress. **(A)** (a-d) represent the strains with different concentrations of hydrogen peroxide. Red highlights in the scan plot indicate specific peaks of o-vanillin. **(B)** Under different hydrogen peroxide concentration stresses area map of o-vanillin peak. **(C)** Survival data of cells treated with o-vanillin under oxidative stress. *p* < 0.05(*); 0.01 < *p* < 0.05(**); *p* < 0.01(***); *p* < 0.0001 (****).

*S. cerevisiae* that had been subjected to various hydrogen peroxide treatments underwent GC-MS analysis. The peak area normalization method was used to compute the relative amounts of each chemical component in the samples, and the retention durations of the metabolites were compared to the mass spectrometry data. The yeast metabolites under stress from hydrogen peroxide were o-vanillin, as shown in Figure 9A. The R32C mutant strain had a higher o-vanillin content than wild-type BY4741 but less than that treated with H_2_O_2_, and the R32C mutant strain treated with H_2_O_2_ had the highest content of o-vanillin (Figure 9B). O-vanillin is a compound that can be used to study oxidative stress and antioxidant responses. In some studies, o-vanillin has been used as an antioxidant or a compound that increases the levels of endogenous antioxidants such as glutathione (Paul et al., 2021).

To clarify that increased o-vanillin levels may be protective for yeast under oxidative stress, cell survival data after treatment with o-vanillin were measured. The results are shown in Figure 9C, where the survival of both WT and R32C cells treated with o-vanillin under 1.75 mM H_2_O_2_ was higher than that of the untreated ones, suggesting that o-vanillin has a protective effect on the cells under oxidative stress conditions.

### Real-time fluorescence quantitative PCR combined with GC-MS analysis

In this study a combination of real-time fluorescence quantitative PCR (polymerase chain reaction) and GC-MS (gas chromatography–mass spectrometry) analytical methods were used.

The transcript levels were quantified using real-time fluorescence quantitative PCR analysis. Total RNA was isolated from the cells and then reverse transcribed into complementary DNA (cDNA). The resulting cDNA samples were subjected to real-time PCR using specific primers for the target transcripts of interest. Furthermore, to analyze the metabolite profiles, the cells were subjected to GC-MS analysis. This technique separates and identifies the metabolites present in the sample based on their retention times and mass spectra.

**Figure 10:**
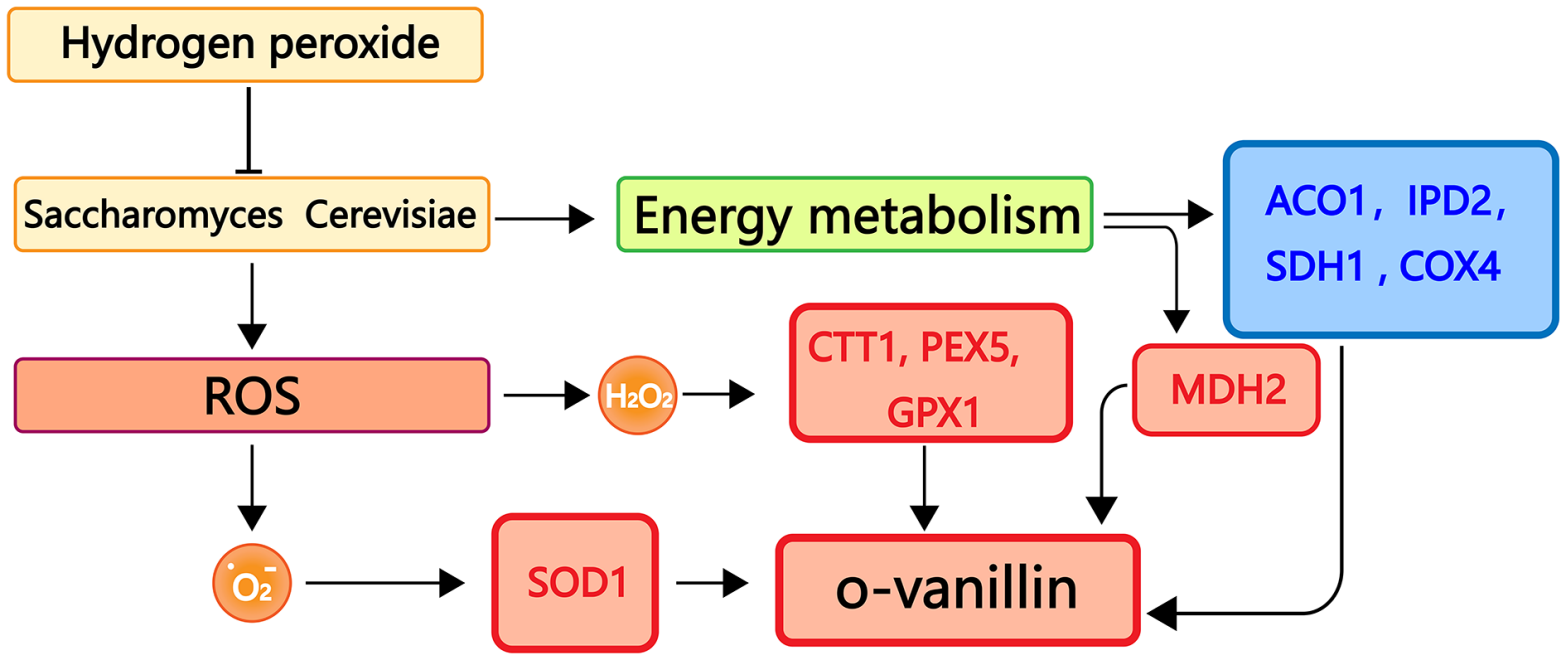
Real-time fluorescence quantitative PCR, GC-MS binding analysis graph. Red represents up-regulated genes, blue represents down-regulated genes.

To better understand the connections between genes and metabolites, we carried out experimental assessments between the transcript levels of genes involved in antioxidant and energy metabolism and metabolites in *S. cerevisiae*. The antioxidant system is essential for protecting cells from harmful outside influences and scavenging oxygen free radicals. As shown in Figure 10, oxidative stress can lead to an increase in the amount of reactive oxygen species in *S. cerevisiae*, which in turn causes an increase in the amount of superoxide anion radicals and H_2_O_2_ in the organism and an increase in the expression levels of antioxidant-related genes *SOD1*, *CTT1*, *PEX5*, and *GPX1*. Conversely, the transcriptional expression levels of genes related to energy metabolism *ACO1*, *IPD2*, *SDH1*, and *COX4* decreased. *MDH*2 gene transcript expression levels, however, were markedly up-regulated. The level of o-vanillin rose under oxidative stress, according to the examination of *S. cerevisiae* metabolites by GC-MS, and o-vanillin is crucial for the body’s antioxidation process. This encouraged the cells to produce more o-vanillin to combat the adverse reaction and prevent damage to the organism.

Numerous illnesses such as neurodegenerative diseases are brought on by oxidative stress. We can offer theoretical direction for comprehending the development of oxidative stress-related disorders and creating treatment approaches by investigating the molecular regulatory systems and metabolic alterations of cells under oxidative stress.

## Discussion

There is growing evidence that succinate dehydrogenase (SDH) is a source and regulator of reactive oxygen species (ROS). Both losses of the function of SDH can lead to intracellular production of ROS with associated effects on the development of pathophysiological conditions, cancer and neurodegenerative diseases (Hadrava Vanova et al., 2020). The addition of ROS concentrations can initiate different cellular actions. Physically, they support signaling pathways involved in cell growth and protection, while their high levels lead to cell damage and death. The balance between ROS production and ROS clearance needs to be strictly regulated (Dröge, 2002).

SDH mutations both lead to increased ROS production in cancer and neurodegenerative diseases (Yankovskaya et al., 2003; Bardella et al., 2011; Iverson et al., 2012). Oxidative stress is a key factor in the emergence of disease and many diseases are associated with oxidative stress. In this study, Exogenous addition of H_2_O_2_ mimics the oxidative stress environment in humans. Compare the response of R32C mutant and wild-type cells to oxidative stress sensitivity. A yeast model was constructed based on the R32C mutant strain of the succinate dehydrogenase *SDH2* gene, in order to confirm the mutation’s pathogenic significance—whether it causes succinate dehydrogenase SDHB-related neurodegenerative diseases in humans associated neurodegenerative diseases.

Recently, Mayr et al. (2010) discovered a patient with a homozygote (Y12C) mutation in the nuclear gene ATP5E, which codes for the ATP synthase subunit. This mutation has been demonstrated to have a considerable impact on the assembly of the human ATP synthase complex, in contrast to its negligible impact on ATP synthase activity in yeast, indicating that the biogenesis of the complex is significantly different. A novel succinate dehydrogenase subunit B gene germline missense mutation (C191Y) found in individuals with glomerular tumors has been the subject of functional studies in a yeast model. These studies suggest the C191Y SDHB mutation suppresses SDH enzyme activity, increasing ROS formation and mtDNA mutability in our yeast model (Goffrini et al., 2009; Panizza et al., 2013) results of study demonstrate that the yeast is a good functional model to validate the pathogenic significance of SDHB missense mutations.

The reduced SDH enzyme activity of the R32C mutant strain suggests that mutations in the gene encoding the SDH2 complex reduce its catalytic activity, leading to reduced respiratory rate and cellular damage, which in turn affects cellular activity. The R32C mutation may have an effect not only on the cellular localization of SDH2, but also on the protein abundance of SDH2, which is normally located in mitochondria and functions in the mitochondrial respiratory chain complex II. Mutations may result in aberrant localization of SDH2, preventing it from correctly localizing to mitochondria or interacting abnormally with other organelles (Garcia Seisdedos et al., 2022). The mutated protein abundance may be different from that of the wild-type (WT) protein (Pastor et al., 2009). Based on a three-dimensional structural model of succinate dehydrogenase, we know that the arginine residue establishes interactions with the other amino acids of the SDH2 subunit, and that replacing the residue with cysteine alters these interactions. So we hypothesized that the changes in these interactions may be related to the development of neurodegenerative diseases.

While our study provides insights into the ultrastructural changes observed in the cells, it is important to consider potential confounding factors that could contribute to these alterations. The treatment of cells with H_2_O_2_, known to induce oxidative stress, could potentially trigger secondary effects that contribute to the observed ultrastructural changes. For example, H_2_O_2_ treatment might lead to osmotic stress or alterations in pH, which can independently impact cellular morphology and organelle integrity (Erkmen, 2021).

In fact, although the mutation itself induces oxidative stress, according to experimental results, R32C mutants have a diminished ability to combat oxidative stress when exposed to H_2_O_2_ stress. Here are some possible reasons: Dysregulation of redox signaling pathways: R32C mutations may disrupt redox signaling pathways, which play a key role in coordinating cellular responses to oxidative stress. Increased ROS production: Although the R32C mutation induces oxidative stress, the mutation may also result in increased ROS production under basal conditions. Higher ROS levels may have burdened the antioxidant defense system, making the mutant cells more susceptible to additional oxidative stress induced by H_2_O_2_. Impaired Repair Mechanisms: R32C mutations may affect repair mechanisms for oxidative damage, such as DNA repair or protein quality control systems. Impairment of these repair processes may lead to the accumulation of damaged molecules, further reducing the ability to cope with exogenous oxidative stress.

The primary method for supplying energy is the tricarboxylic acid cycle. Mutations in TCA cycling enzymes such as SDH predispose carriers to Neurodegenerative diseases. Our findings suggest that R32C mutations lead to significant dysregulation of key stress-responsive genes, including up-regulation of genes involved in antioxidant defense and down-regulation of genes related to energy metabolism. This dysregulation suggests that the normal transcriptional control of stress-responsive genes is disturbed, which may lead to cellular dysfunction and susceptibility to oxidative damage. *MDH2* was significantly upregulated in this study. The following are some potential causes: (1) Hydrogen peroxide can impair the integrity and function of *S. cerevisiae* cell membranes, which indirectly inhibits energy metabolism; (2) hydrogen peroxide triggers a burst of reactive oxygen species in *S. cerevisiae*, which damages DNA and impairs mitochondrial function among other things, inhibiting energy metabolic pathways; and (3) (Carere et al., 2014) indicated that the decrease in the amount of ATP in *Clostridium thermocellum* resulted in a deficiency of NADPH and NADP+ and as a compensation, the cells increased the activity of MDH, resulting in more NADPH production. After hydrogen peroxide treatment, the tricarboxylic acid cycle was broken.

O-vanillin is a potent ROS scavenger in several antioxidant assays. It implies that more o-vanillin is promoted under oxidative stress, improving cellular resistance to negative effects. According to Li et al. (2022) O-vanillin disturbed the ultrastructure of mitochondria, and qRT-PCR dramatically changed the relative expression levels of enzyme genes related to the TCA cycle. As a result, o-vanillin is linked to energy metabolism. Since SDHB is an important component of mitochondria, its function is crucial for cell growth and development. However, *SDHB* mutations alter metabolites (Figure 9A), which may modulate the innate immune response and determine the pro-tumor immune response *in vivo* (Macias-Ceja et al., 2019). For example, lactate and succinate have been reported as pro-tumor metabolites that regulate tumor-associated macrophage polarization, a common feature of malignant solid tumors. Indeed, in *SDHB* mutant cells, altered metabolites may lead to different anti-tumor vs. pro-tumor responses, which overcomes the defect of *SDHB* dysfunction in cell growth and development.

Our research demonstrates that yeast is an appropriate and functional model for testing the pathogenic potential of new missense *SDHB* mutations. Furthermore, conservation analysis throughout evolution could provide additional information for functional testing to determine the pathogenicity of mutations.

Our results indicate that the R32C *SDH2* mutation inhibits cell development and growth, resulting in significant accumulation of ROS formation, increased antioxidant activity and secondary metabolites in the yeast model. While other yeast models for SDH mutations exist, we establish a novel specific model of the VUS R38C and interpret the biological mechanism of cellular oxidative stress. These findings contribute to our understanding of the mechanisms behind the onset of neurodegenerative diseases and highlight the yeast model as an effective method for investigating the potential pathogenic significance of novel mutations and uncommon polymorphisms in SDH.

## Data availability statement

The original contributions presented in the study are included in the article/Supplementary material, further inquiries can be directed to the corresponding authors.

## Author contributions

JZ, DW, and HC: investigation. HC: project administration. DW: resources. HZ: supervision. JZ: writing—original draft. SS, SL and LL: writing—review and editing. All authors contributed to the article and approved the submitted version.

## Footnotes

^1^ http://chromium.liacs.nl/lovd_sdh
