# Supplementary material for: Unraveling the functional consequences of a novel germline missense mutation (R38C) in the yeast model of succinate dehydrogenase subunit B: insights into neurodegenerative disorders
Source: Front Mol Neurosci. 2023 Sep 28;16:1246842. doi: 10.3389/fnmol.2023.1246842 (PMC10568460; doi:10.3389/fnmol.2023.1246842)
Supplement: Supplementary file 1 [file Data_Sheet_1.docx]

**Supplementary Figure 1**


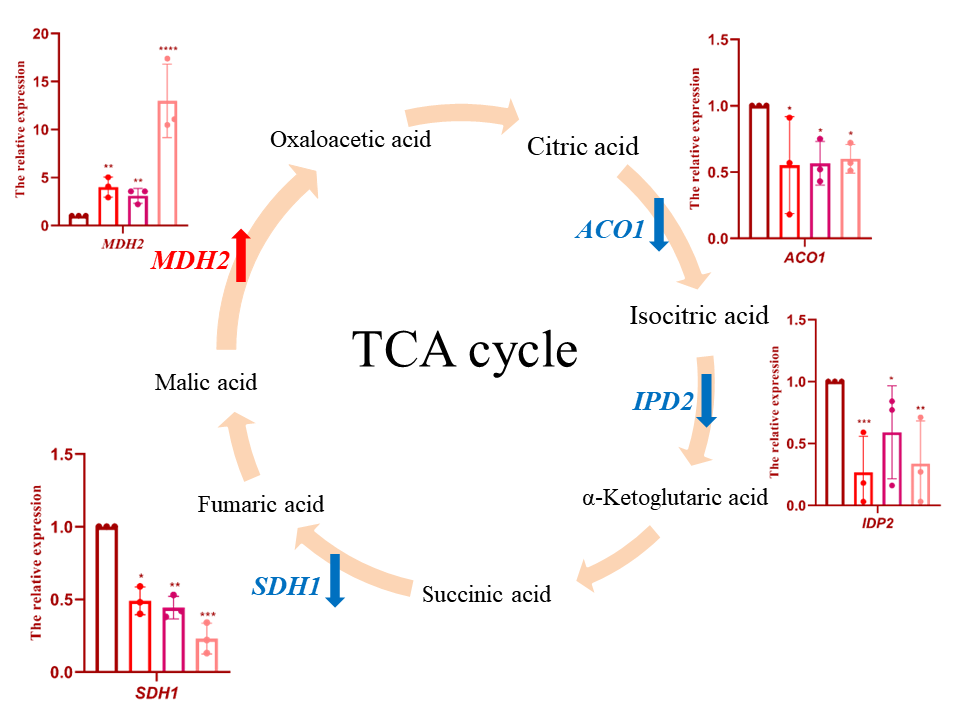


Distribution of genes related to energy metabolism—TCA cycle. Red represents up-regulated genes, blue represents down-regulated genes. *p*<0.05(*); 0.01<*p*<0.05(**); *p*<0.01(***);*p*<0.0001(****).
